# PPARɑ variant V227A reduces plasma triglycerides through enhanced lipoprotein lipolysis

**DOI:** 10.1016/j.jlr.2025.100806

**Published:** 2025-04-15

**Authors:** Lauren F. Uchiyama, Gabriel P.M. Ordonez, Khoi T. Pham, John P. Kennelly, Maykel López Rodríguez, Lany Tran, Peter Tontonoz, Alexander Nguyen

**Affiliations:** 1 Department of Pathology and Laboratory Medicine, University of California, Los Angeles, CA; 2 Department of Biological Chemistry, University of California, Los Angeles, CA; 3 Vatche and Tamar Manoukian Division of Digestive Diseases, Department of Medicine, David Geffen School of Medicine, University of California, Los Angeles, CA

**Keywords:** lipolysis and fatty acid metabolism, lipoprotein lipase, liver, PPARs, triglycerides

## Abstract

Human single nucleotide variants in peroxisome proliferator-activated receptor-ɑ (PPARɑ) have been associated with beneficial metabolic phenotypes, yet their specific effects on metabolic gene expression are not well defined. Here, we developed a mouse model of a human PPARɑ variant encoding a substitution of valine for alanine at position 227 (V227A) to explore the role of this variant on systemic metabolism. Substitution with this variant in mice reduced plasma triglycerides, without altering body mass or liver lipid accumulation, consistent with phenotypes observed in human cohorts. Gene expression analysis revealed that the V227A variant enhances *Ppara* target gene expression in mouse liver, consistent with the effects of synthetic PPARɑ agonist treatment. Notably, V227A increased hepatic expression of *Lpl*, the predominant enzyme responsible for circulating triglyceride hydrolysis. Further characterization revealed that heart tissue from variant mice exhibited increased *Lpl* expression and triglyceride hydrolysis activity, suggesting that V227A enhances cardiac triglyceride clearance. These findings validate human observational studies and clarify the physiological impact of the V227A PPARɑ variant on plasma triglycerides.

Peroxisome proliferator-activated receptor-ɑ (PPARɑ) is a ligand-activated nuclear hormone receptor that regulates tissue and systemic lipid metabolism. Under fasting conditions, ligand-bound PPARɑ promotes transcription of genes involved in peroxisomal and mitochondrial β-oxidation, fatty acid transport, lipoprotein catabolism, and ketogenesis. The importance of PPARɑ in liver has been well characterized in mouse models. Loss of hepatocyte *Ppara* leads to hepatic steatosis due to impaired fatty acid catabolism (1), while activation improves steatohepatitis in models of metabolic dysfunction-associated steatotic liver disease (MASLD) (2). *Ppara*-deficient mice exhibit increased plasma triglycerides with increased hepatic VLDL secretion as a major contributor in female mice (3, 4). PPARɑ agonist treatment reduces plasma triglycerides through both reduced VLDL secretion and enhanced circulating triglyceride clearance (5). Given the use of PPAR agonists in the management of clinical hypertriglyceridemia and in clinical trials for MASLD-associated steatohepatitis and fibrosis (6), understanding the molecular regulation of PPARɑ transcriptional activity may provide opportunities for control of metabolic dysfunction.

Coding variants in human PPARɑ resulting from non-synonymous single nucleotide polymorphisms (SNPs) have been implicated in altered lipid metabolism (7). The rs1800234 T > C variant, encoding alanine instead of valine at position 227 (V227A), is one of the most prevalent coding variants. It exists in Asian and Latino populations with an allele frequency of approximately 4% (www.ncbi.nlm.nih.gov/snp/docs/gsr/alfa/). This variant has consistently been associated with reductions in serum triglycerides and cholesterol in Asian cohort studies (8, 9, 10). An additional study found V227A to be associated with lower prevalence of non-alcoholic fatty liver disease (NAFLD) and obesity as measured by body-mass-index, hip circumference, and waist-to-hip ratio (11). Whether these clinical observations are correlative or causally related has not been clarified. Position 227 in the PPARA protein is located within the hinge region between the DNA binding and ligand-binding domains, and this region is known to be a site for post-translational modification and co-factor binding (12). One study using an overexpression HeLa cell culture model demonstrated that expression of PPARA V227A impairs transcriptional activation of target genes through enhanced recruitment of the nuclear receptor corepressor (NCoR) (13). While this in vitro result suggests that V227A is a loss-of-function mutation, observed human phenotypes are more consistent with V227A being an activating mutation in the context of known PPARA function. The role of PPARA variants in in vivo models has not yet been explored.

Here, we generated a mouse model of the V227A variant through germline base editing of the *Ppara* locus. Base editing allows for specific introduction of a germline variant without notable off-target effects. We demonstrate that this single PPARɑ amino acid substitution impacts physiologic lipid metabolism through a reduction in circulating triglyceride levels. V227A was associated with increased expression of PPARɑ target genes, indicating an activating mutation. Specifically, this variant enhanced *Lpl* transcription in both liver and heart, likely contributing to enhanced plasma triglyceride clearance. Our findings provide insight into the impact of a human PPARɑ coding variant on tissue and systemic lipid metabolism.

## Materials and Methods

### Mice

Custom CRISPR sgRNA using protospacer sequence TATGACCCGGGCCTT was purchased from IDT. Full sequence is in Supplemental Materials. To generate mRNA encoding optimized adenine base editor, plasmid NG-ABEmax (Addgene #124163, gift from David Liu) was linearized with BbsI and purified using PCR Purification Kit (Qiagen). In vitro transcribed RNA was generated with linearized plasmid using mMESSAGE mMACHINE T7 Ultra kit (Ambion) and purified using MEGAclear Kit (Ambion). Mice were generated by the UCSD Transgenic Core Facility. C57BL/6NHsd (Envigo) zygotes were injected with mRNAs encoding NG-ABEmax and targeting sgRNA. Mice were initially genotyped by Sanger sequencing. A heterozygous founder mouse was obtained and backcrossed to C57BL/6NHsd mice. Genotyping strategy for the mouse colony was developed by performing SrfI (New England Biolabs) enzymatic digestion overnight on ∼400 bp PCR product containing mutation in the middle of the amplicon. Digest was separated on agarose gel electrophoresis. Primer sequences are in Supplemental Materials. Mouse experiments were performed on adult mice aged 12–14 weeks. Mice were housed in a temperature-controlled room (22°C with humidity approximately 50%–65%) with a 12-h light–dark cycle and under pathogen-free conditions. Mice were bred as heterozygotes. Only wild-type and homozygous mutant mice were used for experiments to maximize effect size. Mice were fed a chow diet (PicoLab Rodent Diet 20, 5053) or Western Diet (Research Diets, D12079B) unless otherwise stated. GW7647 (Cayman) was prepared in 0.5% hypromellose and gavaged at 16 and 21 h prior to tissue harvest. Fenofibrate diet (Research Diets, D22062001) contained 0.2% fenofibrate and 10 kcal% fat. All animal experiments were approved by the UCLA Animal Research Committee (protocols 1999-131 and 2003-166). For 6-h fasting experiments, mice were fasted from 8am to 2pm. For 16-h fasting experiments, mice were fasted from 8pm to 12pm unless otherwise indicated. Lipase activity and triglyceride uptake experiments were performed on 16-h fasted mice.

### Cloning

To generate sgRNA expression plasmid, phosphorylated primers containing protospacer sequence (GTATGACCCGGGCCTT) were used to amplify pFYF1320 EGFP Site#1 (Addgene #47511) using KOD Hot Start polymerase (Millipore). PCR products were subject to *DpnI* treatment (NEB), DNA purification (QIAPrep columns, Qiagen) and T4 ligation (NEB). Primer sequences are in Supplemental Materials.

### Cell culture

Hepa1-6 cells (ATCC) were cultured in Dulbecco’s modified Eagle’s medium (DMEM) with 10% fetal bovine serum (FBS) and antibiotics at 37°C and 5% CO_2_. NG-ABEmax and cloned sgRNA expression plasmids were used for transient transfections. Lipofectamine 2000 (Invitrogen) was used for plasmid transfections after which cells were transiently selected using puromycin (1ug/ml) for 24 h. Dilution cell cloning was performed on surviving cells. DNA was isolated and purified from clonal populations (QIAPrep columns, Qiagen) and subject to PCR amplification prior to Sanger sequencing.

### Lipid measurements

Tissues were homogenized followed by lipid extraction and quantitation as previously described (14). Cholesterol (WAKO, NC9138103), triglycerides (Sekisui, 36-100-4169), and non-esterified fatty acids (WAKO, 991–34891) were measured from plasma and lipid fraction from tissues using the indicated colorimetric assay.

### VLDL secretion

Adult male mice were fasted for 16 h overnight (4pm-8am) followed by intraperitoneal poloxamer-407 (Sigma) injection and assessment of VLDL secretion as previously described (14).

### Gene expression analysis

For qRT-PCR, mouse liver tissue was subject to RNA extraction, cDNA synthesis, and qRT-PCR as previously described (14). qRT-PCR primers are described in Supplemental Materials. For RNA sequencing, RNA from frozen liver tissue was homogenized and purified using the RNeasy Mini Kit (Qiagen). Stranded poly-A library preparation (Illumina) and paired-end sequencing on NovaSeq X Plus were performed by UCLA Technology Center for Genomics & Bioinformatics. Fastq files were uploaded to the Galaxy web platform, and the public server at usegalaxy.org was used to analyze the data (15). Reads were trimmed using Trimmomatic (16), assessed for quality using FastQC (https://www.bioinformatics.babraham.ac.uk/projects/fastqc/), and aligned to GRCm38/mm10 using RNA STAR (17). Aligned reads were counted using FeatureCounts (18). EdgeR (19) was used for differential expression analysis. For GSEA, GSEA 4.3.3 (20) was downloaded and used locally. Genes were ranked using sign x -log_10_ (*P*-value). For Enrichr, top upregulated genes with *P*-value < 0.05 (39 genes) were used as input in Enrichr (21). GSE118789 was used to assess fasting and fed mouse liver gene expression (22). GSE8396 and GSE154275 were used to assess PPARα agonist-treated and *Ppara* KO liver gene expression (23, 24). These datasets were analyzed on the usegalaxy.org server as above.

### Chromatin immunoprecipitation-qPCR

Chromatin was prepared from frozen liver tissue samples from wild-type and homozygous mutant mice (85–90 mg of frozen liver). Tissue samples were minced with a razor blade on dry ice and then crosslinked in 1% formaldehyde in PBS for 10 min with rotation at room temperature. Glycine (125 mM) was added to stop crosslinking with rotation for 5 min. Subsequently, the samples were washed in ice-cold PBS supplemented with protease inhibitor cocktail (Active Motif). The samples were then lysed in 1 ml of ChIP cell lysis buffer (10 mM Tris-HCl, pH 8.0, 10 mM NaCl, 3mM MgCl_2_, 0.5% NP-40) supplemented with protease inhibitor and homogenized with a Dounce homogenizer. Released nuclei were precipitated by centrifugation and lysed in 700uL of ice-cold ChIP nuclear lysis buffer supplemented with protease inhibitor (1% SDS, 5mM EDTA, 50 mM Tris-HCl, pH 8.1). Chromatin was in a Covaris LE220 E220 Focused Ultrasonicator (Covaris) with 12 cycles (30 s on/30 s off). Chromatin immunoprecipitation was performed as described before (25). 10–25 μg of chromatin was immunoprecipitated with 2–4 μg of PPARα antibody (Santa Cruz Biotechnology, SC398394X). Promoter and enhancer regions for qPCR were selected based on previously reported genomic regions with PPARα binding by ChIP (26, 27). Primer sequences for target and control regions are presented in Supplemental Table S1. Negative genomic region (promoter of *Rpl35*) displaying no previously reported PPARα binding was used to normalize across all samples in the qPCR calculations, and the data is expressed as fold-change over input.

### Measurement of lipase activity

Adult female mice were fed Western diet for 4 weeks prior to 16-h fast. Heart and liver tissues were harvested and processed as previously described (28). Briefly, tissues were homogenized in assay buffer (25 mM NH_4_Cl, 5 mM EDTA, 0.01% SDS, 45 U/ml heparin, 0.05% zwittergent detergent (3-(N,N-Dimethylmyristylammonio)-propanesulfonate), and Halt Protease Inhibitor cocktail (Thermo Fisher)) and incubated for 30 min on ice. Homogenates were centrifuged at 12,000×g for 10 min at 4°C, and the supernatants were isolated and re-centrifuged under the same settings. BCA assay (Pierce, Thermo Fisher) was used to assess protein concentration. Samples were analyzed using Lipoprotein Lipase (LPL) activity commercial kit (Cell Biolabs, Inc., #STA-610) using manufacturer’s instructions. Sample dilution curve was used to account for background signal and linear quantitation.

### ^3^H-labeled chylomicron tissue uptake

Wild-type mice were fasted for 16 h (4pm-8am) and then gavaged with 150μCi of [9,10-^3^H(N)]-Triolein (American Radiolabeled Chemicals, Inc.) suspended in olive oil. After 2 h, mice were sacrificed, and blood was collected by cardiac puncture for plasma isolation in EDTA tubes. Blood was centrifuged at 2,000×g for 10 min at 4°C to pellet blood cells. Plasma was combined, placed in ultracentrifuge tubes, and overlaid with PBS. Plasma was subject to centrifugation at 100,000×g for 30 min at 12°C using SW55Ti rotor (Beckman Coulter). Chylomicron layer on top was removed and resuspended in PBS for immediate use and confirmation of ^3^H counts by scintillation counts. ^3^H-labeled chylomicrons were administered by tail vein injection into male mice fed a Western diet for 4 weeks prior to a 16-h fast. Retro-orbital eye bleeds were performed to assess the clearance of chylomicrons from circulation. At 15 min, mice were sacrificed, perfused with cold PBS, and then subjected to harvesting of tissues. Tissues were dissolved in 1N NaOH overnight prior to the addition of Ultima Gold Liquid Scintillation Cocktail (Revvity) and assessed for ^3^H counts.

## Results

**Fig. 1:**
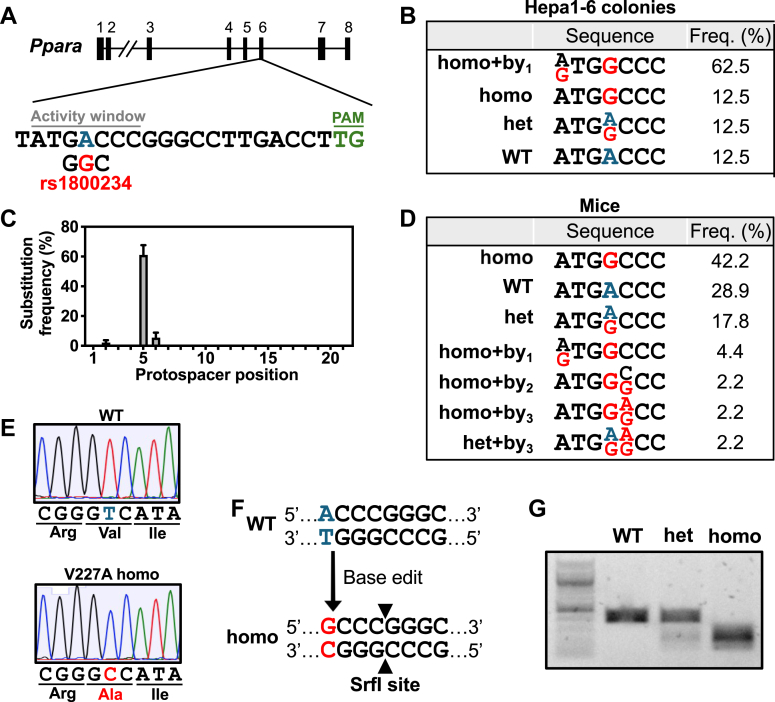
Generation of *Ppara* V227A mouse model. A: Schematic representation of the gRNA target site in the *Ppara* gene locus at exon 6. The protospacer sequence is shown with activity window nucleotides and PAM sequence indicated. Wild-type adenine being targeted is blue, and base-edited change corresponding to indicated human SNP is red. B: Clonal populations derived from Hepa1-6 cells transfected with base editing plasmids (n = 8) were sequenced with activity window mutations and frequency shown. C: Adult mice derived from mouse zygotes microinjected with base editing mRNAs (n = 45) were sequenced, and total substitution frequency along protospacer sequence was calculated. D: Activity window mutations and frequency from (C) is shown. E: Representative Sanger sequencing traces from V227A colony mice with indicated codons and corresponding amino acids. F: Genotyping strategy using *SrfI* restriction enzyme site generated following successful base editing. G: Representative agarose gel with indicated genotypes is shown.

Analysis of the mouse *Ppara* genetic locus revealed that valine 227 is conserved between humans and mice. To study the physiologic impact of the V227A variant, we utilized CRISPR/Cas9-mediated base editing to generate a mouse encoding a V227A germline substitution. Survey of the neighboring nucleotide sequences revealed a suitable high-fidelity adenine base editor with a compatible PAM sequence (NB-ABEmax) and guide RNA protospacer sequence with an activity window appropriately positioned to induce the desired point mutation encoding alanine at position 227 (Fig. 1A). Base editing was designed to be performed on the antisense strand with the A-to-G base edit corresponding to a T-to-C change on the sense strand, which is identical to the rs1800234 single-nucleotide polymorphism found in humans. Transfection of mouse hepatoma cell line Hepa1-6 with plasmids encoding guide RNA and base editor led to predominantly homozygote introduction of the V227A variant at the endogenous locus, validating the suitability of these tools to perform the intended base edit (Fig. 1B). In cells, the intended homozygous mutation was often accompanied by a bystander mutation within the activity window caused by the A-to-G adenine base editing activity. Bystander mutations are more frequent with high doses of base editing components and transfection of plasmid DNA transfection (29). The bystander effect was anticipated to be minimized when performing in vivo base editing of mouse zygotes.

Generation of the V227A mouse strain was performed by microinjection of zygotes with mRNAs encoding guide RNA and the base editor. Of the 45 zygotes implanted with base editing RNAs, 19 mice demonstrated a homozygous mutant genotype (Fig. 1C, D), consistent with previously described high editing efficiency in mouse zygotes (30). Less bystander editing occurred in mice (11%). No proximal off-target editing was seen within 200 base pairs of target nucleotide in all mice (data not shown). Prediction tools (31) to assess for distal off-target editing revealed no potential high-scoring exonic editing and only two low-scoring exonic regions that possessed 3 mismatches. PCR amplification followed by Sanger sequencing of these two exonic regions encoding *Sptbn2* and *Rbm27* did not show any mutations (data not shown). The mouse colony was maintained via heterozygous crosses and yielded expected Mendelian ratios. Sanger sequencing confirmed the presence of the A-to-G mutation that yielded the V227A variant (Fig. 1E). Genotyping of progeny from the mouse colony was subsequently performed based on the mutant allele generating a restriction enzyme *SrfI* cleavage site (Fig. 1F, G).

**Fig. 2:**
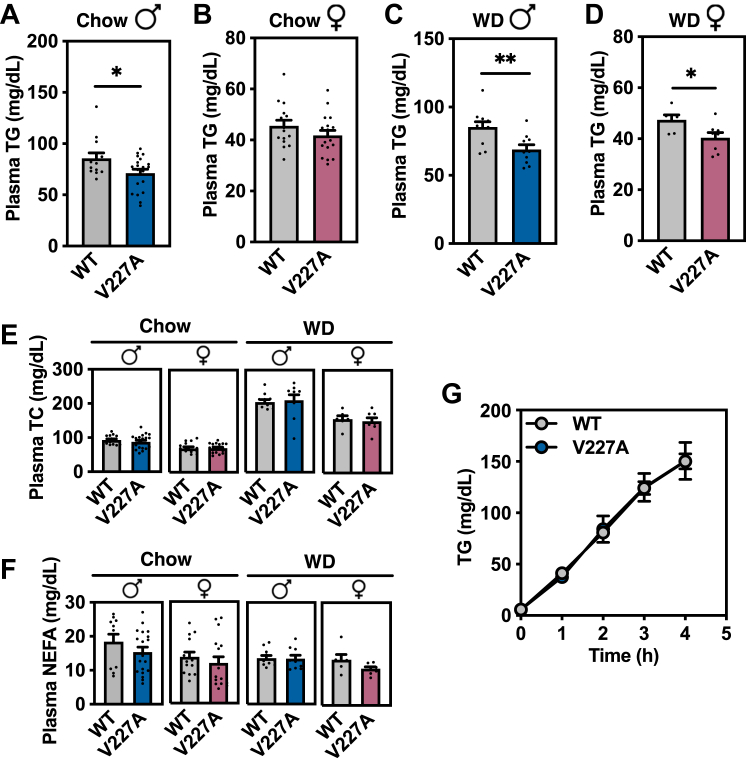
V227A decreases plasma triglycerides. A–D: Plasma triglyceride levels following a 6-h fast were quantified from chow-fed male (n = 13–16 per group) (A), chow-fed female (n = 15–16 per group) (B), Western diet-fed male (n = 10–11 per group) (C), and Western diet-fed female mice (n = 6–8 per group) (D). E: Plasma total cholesterol levels were quantified from mice in (A–D). F: Plasma total non-esterified fatty acid levels were quantified from mice in (A–D). G: 16-h fasted male mice were injected with poloxamer-407 (1 g/kg) followed by blood collection and plasma triglyceride quantitation at indicated times. n = 5 per group. ∗ = *P* < 0.05, ∗∗ = *P* < 0.01 as assessed by two-sided Student’s *t* test. Error bars are SEM.

To explore the impact of this variant on physiology, cohorts of mutant mice were generated under regular chow diet and high-fat Western diet feeding. Wild-type mice were compared to homozygous mutant V227A mice for all studies. We first examined plasma neutral lipid levels because human studies most consistently associate V227A with plasma lipid alterations. Under multiple conditions, mice with the V227A variant exhibited decreased plasma triglycerides following a 6-h fast (Fig. 2A–D), consistent with human observations. Plasma triglycerides were reduced in male mice fed a regular chow diet, and even more prominently reduced in male and female mice fed a high-fat Western diet. Despite females exhibiting decreased plasma triglycerides when fed a Western diet, there was no detectable difference in females fed regular chow, which may be due to lower absolute levels of fasting plasma triglycerides in females and the more modest impact of V227A on chow. There was no difference in plasma non-esterified fatty acids or cholesterol levels regardless of diet type (Fig. 2E, F). While human V227A has been associated with lower serum total cholesterol levels in humans, our mouse model is not well suited for analysis of cholesterol metabolism due to species-specific plasma lipoprotein profiles (the high-density lipoprotein predominance in mice vs. the low-density lipoprotein predominance in humans).

Because fasting plasma triglycerides are carried predominantly by VLDL particles, we investigated the impact of the V227A variant on VLDL metabolism. Triglycerides are packaged into VLDL particles in hepatocytes and secreted into circulation, while triglycerides from circulating VLDL particles are hydrolyzed inside tissue capillaries. Activation of PPARɑ using synthetic agonists strongly reduces VLDL secretion in mice (5), raising the possibility that V227A could impact VLDL secretion. However, variant mice displayed no detectable difference in liver-derived VLDL triglyceride secretion compared to controls under chow-fed conditions (Fig. 2G). These results suggest liver triglyceride secretion is likely not the major contributor to decreased plasma triglycerides in V227A mice.

**Fig. 3:**
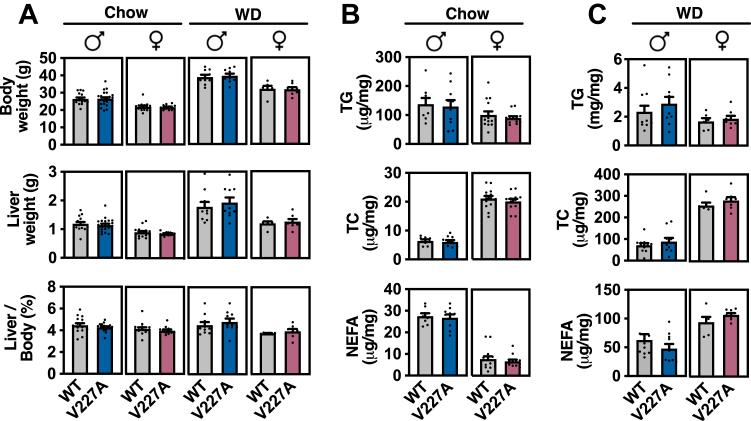
V227A does not alter body mass or liver lipid accumulation. A: Chow-fed male (n = 13–16 per group), chow-fed female (n = 15–16 per group), Western diet-fed male (n = 10–11 per group) and Western diet-fed female mice (n = 6–8 per group) were assessed for body weight, liver weight, and liver-to-body weight ratio following a 6-h fast. B and C: Liver triglycerides, liver total cholesterol and non-esterified fatty acids were quantified from liver tissue harvested from chow-fed mice (B) and Western diet-fed mice (C) indicated in (A). Error bars are SEM.

We next evaluated mice for liver neutral lipid accumulation and the body weight gain, given the reported human V227A association with MASLD and obesity, respectively. Chow-fed male and female variant mice demonstrated no difference in body weight, liver weight, liver-to-body weight ratio, or liver lipids when compared to control mice (Fig. 3A, B). Similarly, variant mice challenged with a high-fat Western diet displayed no differences in body weight, liver weight, or liver lipids compared to that of control (Fig. 3A, C). Despite the importance of PPARɑ in liver lipid homeostasis, these findings suggest that V227A does not impact liver lipid storage in a physiologically meaningful way. Our model does not support a causal role for the V227A variant in the development of obesity or steatotic liver disease.

**Fig. 4:**
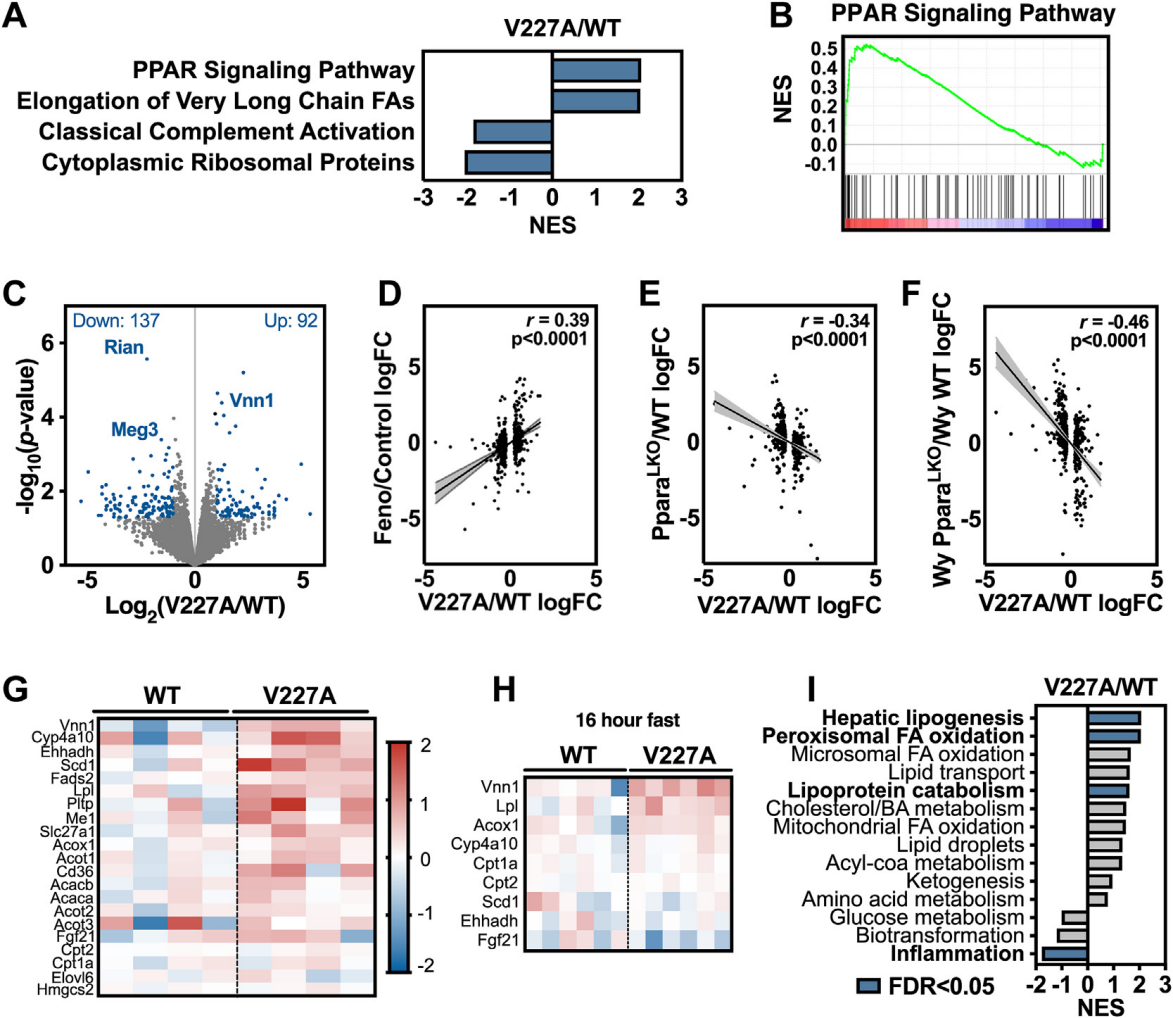
V227A enhances hepatic PPARɑ target gene expression. A: Gene set enrichment analysis with Wikipathways curated gene sets using differential transcript abundance between V227A liver tissue relative to wild-type. Liver tissue subjected to RNA sequencing was obtained from 6-h fasted adult male mice. Gene sets with FDR < 0.05 are shown. B: PPAR signaling pathway gene set enrichment from (A). C: Volcano plot of differentially expressed genes in V227A liver tissue relative to wild-type. Deregulated genes whose *P* < 0.05 and log2 fold-change either > 1 or < −1 are blue. Number of deregulated genes are shown in corners based on direction of change. D-F: Log2 fold-change of top 500 differentially expressed genes were compared between V227A relative to wild-type (x-axis) and indicated comparison (y-axis) using gene expression of 6-h fasted mouse liver tissues from GSE8396. Comparisons include (D) fenofibrate treatment relative to vehicle, (E) liver-specific *Ppara* knock out mice relative to wild-type mice, and (F) Wy-14643-treated liver-specific *Ppara* knock out mice relative to Wy-14643-treated wild-type mice. Linear regression (black line) with 95% CI (grey area) are shown. Pearson correlation coefficient and associated *P*-value are shown. G: Gene expression heat map by log2 fold-change of select PPARɑ target genes in 6-h fasted male wild-type and V227A mutant liver tissue as assessed by RNA sequencing (n = 4). H: Gene expression heat map of select PPARɑ target genes in 16-h fasted male wild-type and V227A mutant liver tissue as assessed by qRT-PCR (n = 6). I: Gene set enrichment analysis with manually curated PPARɑ target gene metabolic pathways using differential transcript abundance between V227A liver tissue relative to wild-type. Gene sets with FDR < 0.05 are labeled in blue with bold text. Genes used for gene sets are listed in Supplemental Materials.

To assess how V227A variant alters *Ppara* molecular activity, we performed RNA sequencing of mutant liver tissue from mice subject to a 6-h fast. Unbiased gene set enrichment analysis (GSEA) identified PPARɑ signaling as the top ranked gene set using Wikipathways gene sets (Fig. 4A, B, Supplemental Fig. S1A). GSEA using top up-regulated genes in Enrichr demonstrated similar enrichment for PPARɑ signaling and lipid and lipoprotein metabolism (Supplemental Fig. S1B, C). Analysis of top deregulated transcripts revealed increased *Vnn1* and decreased *Rian* expression (Fig. 4C), two genes that are similarly altered in PPARɑ agonist-treated mouse livers (24). V227A modestly affected the global transcriptome with relatively few genes being differentially expressed (Fig. 4C), suggesting that the V227A variant mildly enhances expression of PPARɑ gene targets. In mice subject to a 6-h fast, differentially expressed genes in mutant mouse liver tissue correlated positively with genes shown to be altered in livers from mice treated with PPARɑ agonist fenofibrate (Fig. 4D) and negatively with genes altered in liver-specific *Ppara*-null mouse liver tissue with and without agonist Wy-14643 (Fig. 4E, F) (23). These correlations were also seen when compared to 16-h fasted mouse liver tissue following Wy-14643 and fenofibrate treatment (24) (Supplemental Fig. S2A, B) and *Ppara*-null mouse liver tissue with or without agonist treatment (Supplemental Fig. S2C, D). Canonical liver PPARɑ target genes (32) demonstrated consistent upregulation in mutant liver tissue, although to varying degrees (Fig. 4G). For example, *Cyp4a10, Ehhadh* and *Scd1* were upregulated, yet *Elovl6*, *Cpt1a*, and *Cpt2* were relatively unchanged. Under 16-h fasting conditions when PPARɑ is more prominently activated, some of these PPARɑ target genes remained upregulated (Fig. 4H) though to a lesser degree compared to the 6-h fast, suggesting that the modest enhancement of PPARɑ activity by V227A may be less apparent when PPARɑ is fully activated. While a previous study using HeLa cells showed that PPARA V227A overexpression inhibited *Hmgcs2* transcriptional activation (13), we found that *Hmgcs2* transcript levels were unchanged in V227A liver tissue (Fig. 4G). Together, these findings indicate that V227A is an activating variant that enhances expression of PPARɑ target genes during fasting.

Activated PPARɑ coordinates many related yet distinct metabolic pathways that together constitute a physiologic response to fasting. A closer look at the most enriched gene sets from unbiased GSEA revealed enrichment of pathways involved in the synthesis as well as the catabolism of fatty acids (Fig. 4A, Supplemental Fig. S1A). While PPARɑ is well-characterized for its activation of fatty acid oxidation pathways, fatty acid synthesis is believed to be activated as a means to neutralize any excess of incoming adipose tissue-derived fatty acids during fasting (32). To characterize which PPARɑ-driven metabolic pathways are predominantly induced by V227A, we curated metabolic pathway gene sets for use in GSEA based on known PPARɑ target gene cellular activity (32) (Supplemental Table S2). GSEA confirmed that the V227A variant enhances expression of genes involved in hepatic lipogenesis and peroxisomal fatty acid oxidation (Fig. 4I). Activation of fatty acid synthesis and oxidation concurrently may lead to potential futile cycling of fatty acids, rendering any cumulative impact on liver lipid levels potentially inconsequential (Fig. 3B, C). Lipoprotein catabolism genes were also enriched, and inflammation genes were downregulated consistent with known PPARɑ repression of pro-inflammatory genes. While fasting activates primarily fatty acid oxidation and catabolic pathways, and synthetic agonists robustly activate nearly all PPARɑ target gene pathways (Supplemental Fig. S1D), the V227A variant appears to exhibit more selective pathway activation. Considering human metabolic associations, enhanced transcription of lipoprotein catabolism genes would be consistent with a causal role of the V227A variant in the regulation of human plasma lipid levels.

**Fig. 5:**
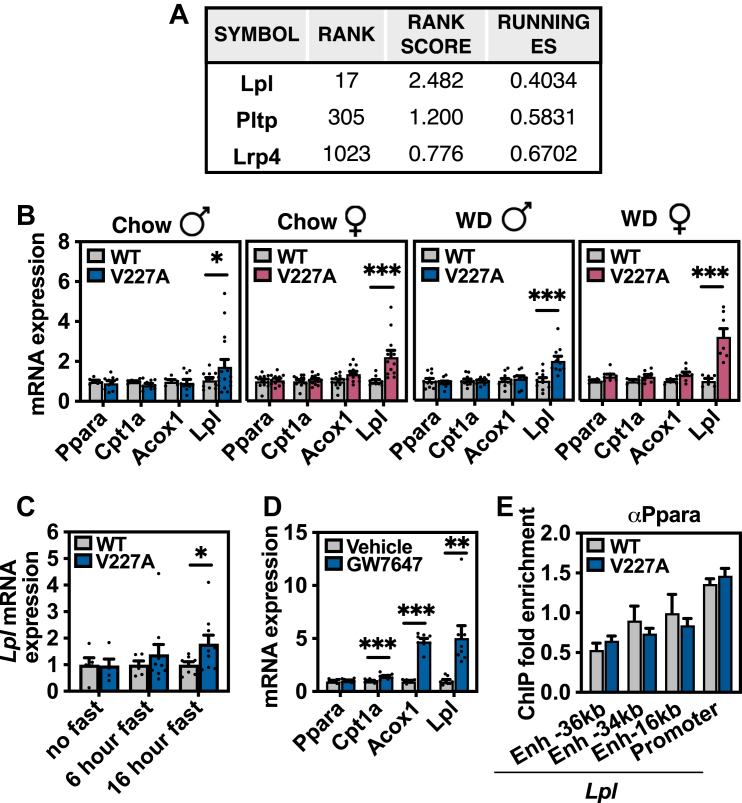
V227A increases *Lpl* transcript abundance in liver. A: Genes that account for GSEA enrichment of lipoprotein catabolism gene set used in Fig. 4I. B: qRT-PCR of PPARɑ target genes using liver tissue from 6-h fasted chow-fed male (n = 11–15 per group), chow-fed female (n = 12 per group), Western diet-fed male (n = 10–11 per group), and Western diet-fed female mice (n = 6–8 per group). C: *Lpl* transcript abundance in liver tissue from male mice with indicated fasting duration as assessed by qRT-PCR (n = 4–9 per group). D: Gene expression in liver tissue from 6-h fasted male mice gavaged with PPARɑ agonist GW7647 (10 mg/kg) as assessed by qRT-PCR (n = 8). E: Ppara binding to *Lpl* enhancer (Enh) and promoter genomic regions in 6-h fasted V227A liver tissue (n = 5) as assessed by ChIP-qPCR. Enrichment is normalized to *Rpl35* promoter region that does not exhibit known PPARɑ binding (27). ∗ = *P* < 0.05, ∗∗ = *P* < 0.01, ∗∗∗ = *P* < 0.001 as assessed by Student’s *t* test. Error bars are SEM.

Review of differentially expressed *Ppara* target genes in V227A livers revealed lipoprotein lipase (*Lpl*), the primary enzyme responsible for clearance of plasma triglyceride, to be strongly and consistently upregulated as the 37th most significant gene out of 18,937 genes, and in the top 0.2% of differentially expressed genes (Fig. 4G, H). *Lpl* upregulation was also the main contributor to the lipoprotein catabolism pathway enrichment by GSEA (Figs. 5A and 4I). The *Lpl* transcript was consistently increased in mutant mouse livers in mice subject to a 6-h fast under all tested conditions regardless of sex or diet as assessed by quantitative RT-PCR (Fig. 5B). *Lpl* is a known *Ppara* gene target (33) and responds to *Ppara*-dependent activation in the liver following prolonged 16-h fasting and pharmacologic PPARɑ agonist treatment in mice (Fig. 5C, D). Multiple human studies suggest *Lpl* is an important target of PPARɑ that decreases fasting triglycerides under agonist treatment (5, 34, 35, 36). Interestingly, V227A did not alter Ppara binding to *Lpl* promoter or upstream enhancer regions as assessed by chromatin-immunoprecipitation (Fig. 5E). While liver-derived *Lpl* has been shown to contribute to the hydrolysis of plasma triglyceride levels in mice (37), *Lpl* transcript and activity are low in adult rodent liver tissue (38, 39, 40, 41). Whether liver lipase activity substantially contributes to the physiologic regulation of plasma triglyceride levels is a topic of debate.

**Fig. 6:**
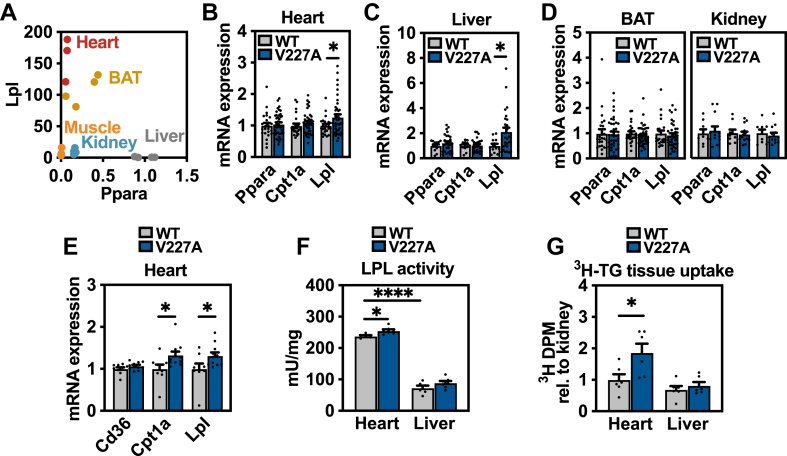
V227A increases cardiac *Lpl* transcript abundance and lipase activity. A: *Ppara* and *Lpl* mRNA expression from indicated tissues obtained from three wild-type mice as assessed by qRT-PCR. Expression is normalized to liver abundance. B: Heart (n = 25–40), (C) liver (n = 13–27), (D) brown adipose tissue (BAT) (n = 25–40) and kidney (n = 9–13) gene expression from 6-h fasted wild-type and V227A mice fed chow diet. E: Heart gene expression from 6-h fasted wild-type and V227A female mice fed Western diet for 4 weeks (n = 9–11). F: LPL activity as assessed using heart and liver tissue harvested from 16-h fasted female mice fed Western diet for 4 weeks (n = 6–7). G: ^3^H disintegrations per minute (DPM) from heart tissue and liver tissue harvested from 16-h fasted male mice 15 min after tail vein inoculation of ^3^H-trigylceride chylomicrons. CPM normalized to kidney CPM. (n = 6). ∗ = *P* < 0.05, ∗∗∗∗ = *P* < 0.0001 as assessed by two-sided Student’s *t* test. Error bars are SEM.

Because V227A exists as a germline variant, we reasoned that other non-hepatic PPARɑ-regulated tissues may also exhibit elevated *Lpl* expression. PPARɑ is expressed predominantly in tissues with high fatty acid oxidation rates such as liver, heart, and brown adipose tissue. Heart and brown adipose tissue both exhibit high *Ppara* expression with abundant *Lpl* transcript, protein, and activity (42, 43) (Fig. 6A). In V227A mice, *Lpl* transcript levels were increased in heart and liver, but were unchanged in brown adipose tissue and kidney (Fig. 6B–D). *Ppara* is known to regulate Lpl activity in mouse cardiac tissue (44). The increase in cardiac *Lpl* transcript was readily detected in V227A variant mice fed a high-fat Western diet (Fig. 6E). Given the nearly 200-fold increase in *Lpl* transcript in the heart, the increase in heart-derived *Lpl* potentially contributes to systemic triglyceride hydrolysis. To test this hypothesis, liver and heart tissue lipoprotein lipase activity from 16-h fasted, Western diet-fed mice were assessed ex vivo. First, wild-type hearts exhibited four-fold higher Lpl activity compared to liver per mg tissue (Fig. 6F). V227A heart tissue demonstrated increased lipoprotein lipase activity compared to that of control, while liver tissue demonstrated no difference between genotypes (Fig. 6F). Finally, to assess lipoprotein lipase activity and triglyceride uptake in vivo, 16-h fasted V227A mice were inoculated with intravenous chylomicrons containing radiolabeled triglycerides. Radiolabeled triglycerides were cleared from circulation within 1 min (data not shown). When tissues were assessed for triglyceride uptake normalized to uptake by the kidney, which expresses low *Ppara* and *Lpl* and no detectable *Lpl* upregulation (Fig. 6A, D), heart tissues from variant mice demonstrated increased uptake, while liver tissue uptake was not different between genotypes (Fig. 6G). Altogether, these data suggest that the metabolically protective PPARɑ variant V227A decreases plasma triglycerides, at least in part, through an upregulation of cardiac *Lpl* transcript.

## Discussion

Previous human observations have demonstrated a strong association between PPARɑ variants and cardiometabolic phenotypes. Whether these associations are correlative or causal has been unclear. By utilizing base editing to generate a PPARɑ variant mouse model, we confirm the human association of the V227A variant with reduced plasma triglyceride levels. We found that V227A is an activating mutation in vivo that selectively enhances transcription of some, but not all, hepatic PPARɑ pathways. Prior studies in rodents using *Ppara* knock-out mice and synthetic agonist treatments have suggested that PPARɑ decreases plasma triglyceride levels via changes in both liver lipoprotein secretion and clearance (4, 5, 45). However, multiple human studies with FDA-approved fibrates support increased clearance through LPL activity as the primary mechanism (5, 34, 35, 36, 46, 47). Our data suggest that mild PPARɑ activation with V227A impacts VLDL catabolism but not secretion (Fig. 2G), which is consistent with these human findings. This mouse model could be used as a tool to characterize selective PPARɑ activation that cannot be captured using complete genetic ablation or synthetic agonism. Our data suggest that mildly increased *Lpl* expression and activity in the heart functionally contribute to the enhanced uptake of circulating triglycerides in V227A mice (Fig. 6F, G).

The V227A model highlights the importance of tissue-specific PPARɑ pathway functions. While V227A enhances liver PPARɑ transcriptional activity, we did not observe prominent effects on liver function. The modest hepatic transcriptional changes did not appear to translate to perturbed lipid metabolism in this tissue-specific context. Our work suggests that the heart may be a major site of increased *Lpl* transcription and activity in V227A mice. While fatty acids contribute to 70% of basal cardiac energy requirements, oxidation of triglyceride-derived fatty acids supplies greater than 90% of cardiac energy needs during the fasting state (48). The heart’s reliance on fatty acids during fasting, coupled with having among the highest *Lpl* expression on a per gram basis (42), may render greater susceptibility to changes in *Lpl* transcript levels. Our findings are supported by previous work that shows cardiac-specific knock-out of *Lpl* led to hypertriglyceridemia due to failed lipoprotein-derived triglyceride clearance (49).

Although we focused on *Lpl* gene expression in the heart, there may be additional cardiac genes that regulate *Lpl* post-translationally that contribute to reduced plasma triglycerides (50). Interestingly, previous work in the heart suggests PPARɑ does not induce *Lpl* gene expression, and instead regulates the transcription of inhibitors (*Angptl3, Angptl4, and Apoc3*), or inducers (*Apoc2*, *Apoa5*) to increase its enzymatic activity (32, 33). While we observed no differences in the gene expression of these regulators in V227A liver tissue (data not shown), transcriptomic characterization of Lpl regulatory genes in the heart could be informative. One study suggested PPARɑ reduces Lpl activity in the heart, though this work was performed in primary rat cardiomyocytes (51). This discrepancy is likely due to differences in vitro, as Lpl activity was observed to double in rat heart tissue following an overnight fast (50).

Other PPARɑ-regulated tissues may also impact plasma triglyceride metabolism. Interestingly, brown adipose *Lpl* transcript levels were not affected by V227A in our mice. These findings indicate that V227A contributes to tissue-specific regulation of PPARɑ activity. V227A did not impact PPARɑ occupancy at the *Lpl* promoter locus in the liver (Fig. 5E), suggesting that changes in DNA binding do not drive the phenotype. Since residue 227 is located within the hinge domain, the V227A variant could potentially alter transcriptional complex stability or dynamics at select target gene promoters. A substitution of valine to alanine alters the residue’s physical size and shape, potentially impacting co-factor binding. How this is mediated molecularly, and which cofactors, contribute will be determined in the future.

Important limitations of our model include species-specific metabolic differences in cholesterol metabolism and response to PPARɑ. Plasma cholesterol cannot be readily compared in our mouse model due to species-specific differences in lipoprotein HDL and LDL abundance. A V227A mouse model with a humanized lipoprotein profile would be better suited to characterize the impact of V227A on cholesterol differences seen in humans. In hepatocytes, PPARɑ synthetic agonist treatment induces massive peroxisome proliferation in rodents, but not humans (32, 52, 53, 54). Our experiments also varied in fasting times as most gene expression studies were performed following a 6-h fast while functional assays were performed following a 16-h fast. PPARɑ is most prominently activated following adipose tissue lipolysis induced by prolonged fasting (55). Because the described human V227A phenotypes were measured under standard clinical fasting practices intended to avoid chylomicron contribution to plasma lipid measurements, we performed our mouse experiments using a 6-h fast to model human collection procedure. V227A-dependent transcriptional changes were detected at both 6-h and 16-h fast (Figs. 4C–H, 5B, C, 6B, C, E). However, while V227A-dependent *Lpl* induction was seen at both fasting durations, V227A did not enhance expression of many other PPARɑ target genes at 16 h when it did so at 6 h. This potentially can be attributed to prominent PPARɑ activation from prolonged fasting may render a subtle transcriptional impact caused by a single amino acid substitution less detectable. Functional triglyceride hydrolysis assays (Fig. 6F, G) were performed following a 16-h fast to maximize PPARɑ-dependent physiological impact. Finally, while our mouse model did not show changes in obesity or hepatic steatosis, these phenotypes may require a different physiologic context with higher sucrose or fat. Future studies addressing these points will be informative.

Whether V227A-mediated plasma triglyceride reduction imparts any long-term cardiometabolic protection is not yet known, but there is clinical interest in promoting LPL activity for the prevention of cardiovascular disease (56, 57). Increased LPL activity via RNA interference of post-transcriptional regulators APOC3 or ANGPTL3 reduced plasma triglycerides in two recent human clinical trials (56, 57). Although it is unclear if a reduction in plasma triglycerides alone mediates cardiovascular protection, both of these trials demonstrated that enhancing LPL activity led to a reduction in remnant cholesterol levels, which are believed to contribute to cardiovascular risk (58). Cardiac-specific PPARɑ overexpression in mice have cardiomyopathy, impaired left ventricular function and insulin resistance, suggesting that excessive activation of the PPARɑ program is maladaptive (59, 60). These studies highlight the value in specific and measured activation of LPL. Should LPL-mediated reduction in plasma triglycerides remain a clinical target, V227A and its transcriptional impact on LPL may expand the opportunities for modes of LPL regulation. Interestingly, human V227A has been associated with reduced total cholesterol with the effect appearing to be driven by the non-HDL fraction (9, 10). Cardiac-specific LPL knock-out mice demonstrated reduced plasma VLDL-cholesterol ester clearance (61), suggesting plasma cholesterol levels may also be regulated by V227A in an LPL-dependent manner. Given the known impact of plasma cholesterol levels on cardiovascular disease, determining whether V227A reduces LDL or remnant cholesterol levels and whether LPL-mediated plasma cholesterol reduction yields any protection from atherosclerosis would be worth future investigation. These studies would help reveal a better understanding into PPARɑ transcriptional selectivity and potentially expand opportunities for clinical lipid control.

## Data availability

Sequencing data have been deposited to GEO under GSE288347. All remaining data are included in the current submission and in the Supplemental Materials.

## Supplemental data

This article contains supplemental data.

## Conflict of interest

The authors declare that they have no conflicts of interest with the contents of this article.
