# Supplementary material for: PPARɑ variant V227A reduces plasma triglycerides through enhanced lipoprotein lipolysis
Source: J Lipid Res. 2025 Apr 15;66(5):100806. doi: 10.1016/j.jlr.2025.100806 (PMC12144439; doi:10.1016/j.jlr.2025.100806)
Supplement: Supplemental Materials [file mmc1.docx]

**SUPPLEMENTAL MATERIALS**

**PPARɑ variant V227A reduces plasma triglycerides through enhanced lipoprotein lipolysis**

Lauren F. Uchiyama^1,2^†, Gabriel Pe Manuel Ordonez^3^†, Khoi Pham^3^, John Paul Kennelly^1,2^, Maykel López Rodríguez^1,2^, Lany Tran^3^, Peter Tontonoz^1,2^, Alexander Nguyen^3^*

^1^Department of Pathology and Laboratory Medicine, University of California, Los Angeles, CA, USA

^2^Department of Biological Chemistry, University of California, Los Angeles, CA, USA

^3^Vatche and Tamar Manoukian Division of Digestive Diseases, Department of Medicine, David Geffen School of Medicine, University of California, Los Angeles, CA, USA

†These authors contributed equally

**Supplemental Table 1. Oligonucleotide sequences**

| **resource type** | **sequences & additional information** |
| --- | --- |
| Cloning primers to introduce sgRNA  into expression vector | forward, 5'- GTATGACCCGGGCCTTGACCT  GTTTTAGAGCTAGAAATAGC  AAGTTAAAATAAGGC -3';  reverse, 5'- GGTGTTTCGTCCTTTCCACAAG -3' |
| Ppara V227A sgRNA for zygote microinjection (IDT) | 5’- GTATGACCCGGGCCTTGACCTGUUUUAGA  GCUAGAAAUAGCAAGUUAAAAUAAGGCU  AGUCCGUUAUCAACUUGAAAAAGUGGCA  CCGAGUCGGUGCUUUU -3’ |
| *Ppara* exon 4 genotyping primers | forward, 5'- AGCAATTCGCTTTGGAAGAA -3';  reverse, 5'- AAGAAGGCAGCAGTTGGTGT -3'  (DNA fragments size after *SrfI* digestion: wild-type, 399bp; mutant, 170bp & 229bp) |
| *Lpl* qPCR primers | forward, 5'- AGGACCCCTGAAGACAC -3';  reverse, 5'- GGCACCCAACTCTCATA -3' |
| *Cpt1a* qPCR primers | forward, 5'- CCGCCAATTCCAAAAAGTAAC -3';  reverse, 5'- CATTTGGTTTGTATCACTAGA -3' |
| *36B4* qPCR primers | forward, 5'- GGCCCTGCACTCTCGCTTTC -3';  reverse, 5'- TGCCAGGACGCGCTTGT -3' |
| *Acox1* qPCR primers | forward, 5'- GCCATTCGATACAGTGCTGTGAG -3';  reverse, 5'- CCGAGAAAGTGGAAGGCATAGG -3' |
| *Ppara* qPCR primers | forward, 5'- GACAAGGCCTCAGGGTACCA -3';  reverse, 5'- GCCGAATAGTTCGCCGAAA -3' |
| *Cd36* qPCR primers | forward, 5'- TTGTACCTATACTGTGGCTAA -3';  reverse, 5'- CTTGTGTTTTGAACATTTCTGC -3' |
| *Vnn1* qPCR primers | forward, 5'- TATGTCTTCCCTGAAGTGTT -3';  reverse, 5'- CCCAGTCCTTCCCATAC -3' |
| *Cyp4a10* qPCR primers | forward, 5'- CTCATTCCTGCCCTTCTCA -3';  reverse, 5'- GTAGTTCGAAGCGGAGCAGT -3' |
| *Ehhadh* qPCR primers | forward, 5'- CGGTCAATGCCATCAGTCCAA -3';  reverse, 5'- TGCTCCACAGATCACTATGGC -3' |
| *Scd1* qPCR primers | forward, 5'- CGAAGTCCACGCTCGATCTC -3';  reverse, 5'- TGTGGGCCGGCATGAT -3' |
| *Fgf21* qPCR primers | forward, 5'- GCTGCTGGAGGACGGTTACA -3';  reverse, 5'- CACAGGTCCCCAGGATGTTG -3' |
| *Cpt2* qPCR primers | forward, 5'- CAGTGTGGGCGAGCTTCAG -3';  reverse, 5'- GAGCTCAGGCAGGGTGACC -3' |
| *Lpl* promoter ChIP-qPCR primer | forward, 5'- CCCCTCCTCTCTGCCTCTAT -3';  reverse, 5'- GGTAACGAGGCTCAACGGTG -3' |
| *Lpl* enhancer -16kb ChIP-qPCR primer | forward, 5'- ACCTGGCTTGCAAAATGACC -3';  reverse, 5'- AGCACTCTACGCCCTTATCT -3' |
| *Lpl* enhancer -34kb ChIP-qPCR primer | forward, 5'- TGCTCTACTGTCCCCAAACA -3';  reverse, 5'- GCCTTGGCAACCGTTACTAG -3' |
| *Lpl* enhancer -36kb ChIP-qPCR primer | forward, 5'- TGTTTGTGTAGCATCTCCTTCTG -3';  reverse, 5'- AAAAGGGGCAGGGAAAGTCA -3' |
| *Rpl35* negative control ChIP-qPCR primer | forward, 5'- TCTGGGACTGTAGGCAAAGG -3';  reverse, 5'- GAAAACCTGCAAAGCTCCCA -3' |

**Supplemental Table 2. *Ppara* target gene metabolic pathway gene sets**

| **Pathway Gene Set** | **Genes** |
| --- | --- |
| Ketogenesis | *Hmgcs2, Fgf21, Bdh1, Acat1, Hmgcl* |
| Hepatic lipogenesis | *Scd1, Fasn, Acaca, Gpam, Fads1, Acacb, Agpat2, Elovl6, Mlycd, Me1, Lpin2, Hsd17b12, Fads2, Agpat3, Agpat5, Gpat4, Dgat1, Elovl5, Elovl7, Mogat1, Scd2, Slc25a10, Srebf1* |
| Mitochondrial FA Oxidation | *Cpt1a, Cpt2, Acaa2, Acadm, Acadl, Acadvl, Acads, Acad8, Acad9, Acad10, Acot2, Etfdh, Hadha, Hadhb, Hadh, Slc25a20, Slc22a5, Txnip, Acot9, Acot10, Cpt1b, Crat, Etfa, Etfb, Decr1, Eci1, Hsd17b10, Hibch, Ucp2, Ucp3* |
| Acyl-CoA metabolism | *Acot12, Acsl1, Acsl3, Acsl5, Acsm3, Acot1, Acot7, Acsl4, Acss2, Fabp1, Fabp2, Fabp3, Fabp4, Fabp5* |
| Lipid transport | *Fabp1, Cd36, Slc27a1, Slc27a4, Slc27a2, Lepr, Adipor2* |
| Lipoprotein catabolism | *Lpl, ApoC3, ApoA1, ApoA2, Apoa5, Pltp, Angptl4, Lipc, Lipg, Lrp4, Pctp, Vldlr, Mttp* |
| Glucose transport and metabolism | *Aqp3, Pdk4, G6pc1, Gk, Aqp7, Aqp9, Fbp2, Gpd1, Gpd2, Gys2, Ldha, Pcx, Pck1, Pdk1* |
| Inflammation | *Cd68, Fgb, Apc2, Birc3, Cebpb, Crp, Cxcl10, Adgre1, Icam1, Ifi47, Igtp, Nfkbia, Il1b, Il1r1, Il1rn, Il1rap, Il6, Il6ra, Il18, Lcn2, Lifr, Ccl2, Ccl3, Mt1, Mt2, Orm2, Orm3, Nfkb1, Pla1a, Saa2, Saa4, Stat1, Stat2* |
| Peroxisomal FA Oxidation | *Acox1, Ehhadh, Abcd2, Abcd3, Acaa1a, Acaa1b, Acot3, Acot4, Acot5, Acot8, Crot, Decr2, Ech1, Hacl1, Hsd17b4, Eci2, Pex11a* |
| Microsomal FA Oxidation | *Aldh3a1, Aldh3a2, Aldh9a1, Cyp4f15, Cyp4x1, Cyp4a10, Cyp4a12a, Cyp4a14* |
| Lipid Droplets | *Plin2, Ces1g, Ces3a, Cidea, Cidec, Plin4, Lipa, Lipe, Mgll, Plin5, Plin1, Pnpla2* |
| Cholesterol/BA metabolism | *Abca1, Abcb4, Abcb11, Abcg5, Abcg8, Cav1, Cyp7a1, Cyp8b1, Cyp27a1, Npc1, Rab9, Scarb2, Slc10a1, Slc10a2* |
| Biotransformation | *Akr1B10, Cyp2c29, Cyp1a2, Cyp2a5, Ephx2, Gsta3, Mgst3, Ugt1a9, Cyp3a11* |
| Amino acid metabolism | *Abat, Acmsd, Agxt2, Arg1, Asl, Ass1, Cbs, Cps1, Cth, Got1, Got2, Gls, Gls2, Gpt, Hal, Hpd, Oat, Odc1, Otc, Pah, Psat1, Tat* |

**Supplemental Figure 1. Additional gene set enrichment analysis analyses.**

**(A-C)** Gene set enrichment analysis with Wikipathways curated gene sets **(A)**, KEGG 2021 Human gene sets **(B)**, and Bioplanet 2019 gene sets **(C)** using differential transcript abundance between V227A liver tissue relative to wild-type. Liver tissue was obtained from 6-hour fasted adult male mice. Gene sets with FDR < 0.05 in **(A)** or p-value < 0.01 in **(B-C)** are labeled in blue. **(D)** Gene set enrichment analysis with manually curated PPARɑ target gene metabolic pathways using differential transcript abundance between the following conditions from left-to-right: Wy-14643 treatment relative to control, fenofibrate treatment relative to control, and 16-fasted relative to fed using data from GSE154275 and GSE118789. Gene sets with FDR < 0.05 are labeled in blue. Genes used for gene sets are listed in supplementary data. NES = normalized enrichment score.

**Supplemental Figure 2. Gene expression correlation with 16-hour fasted mice**

Log2 fold-change of deregulated genes from V227A liver tissue relative to wild-type (p < 0.05 and log2 fold-change either > 1 or <-1) were compared between V227A relative to wild-type (x-axis) and indicated comparison (y-axis) using RNA sequencing of 16-hour fasted mouse liver tissues from GSE154275. Comparisons include **(A)** Wy-14643 treatment relative to vehicle, **(B)** fenofibrate treatment relative to vehicle, **(C)** *Ppara* knockout mice relative wild-type mice, **(D)** Wy-14643-treated *Ppara* knockout mice relative to Wy-14643-treated wild-type mice, and **(E)** fenofibrate-treated *Ppara* knockout mice relative to fenofibrate-treated wild-type mice. Linear regression (black line) with 95% CI (grey area) are shown. Pearson correlation coefficient and associated p-value are shown.
